# 

**Published:** 1883-01

**Authors:** 


January, 1883.

NUMBER 1, VOL. XLVI.

S.G.O. INDEXED

### THE CHICAGO MEDICAL JOURNAL & EXAMINER.

CHICAGO MEDICAL PRESS ASSOCIATION.

AUG. 2D 1875.

CHICAGO:
*PUBLISHED BY THE CHICAGO MEDICAL PRESS ASSOCIATION,*
188 CLARK STREET.

☞ Read the Notice of this Journal among Advertisements.

